# Thyroid Dosimetry and Its Association With Radiation-Induced Hypothyroidism in Head and Neck Cancer Patients Treated With Conformal Radiotherapy: An Observational Study

**DOI:** 10.7759/cureus.78220

**Published:** 2025-01-29

**Authors:** Kushal Sen, Tapas Maji, Debarshi Lahiri, Ranti Ghosh, Debjit Ghosh, Debanjan Chakraborty

**Affiliations:** 1 Radiation Oncology, Chittaranjan National Cancer Institute, Kolkata, IND

**Keywords:** head and neck squamous cell carcinoma, hypothyroidism after cancer radiotherapy, hypothyroidism in carcinoma head-neck, radiation-induced hypothyroidism, thyroid dosimetry

## Abstract

Introduction

Hypothyroidism is a common side effect in head and neck cancer (HNC) patients treated with radiotherapy (RT). Conformal RT reduces the dose to normal tissues while delivering higher doses to tumors. However, hypothyroidism remains a significant toxicity in these patients. This study evaluates the incidence of hypothyroidism, thyroid dosimetric parameters, and their correlation with radiation-induced hypothyroidism in HNC patients treated with conformal RT at a regional cancer center in India.

Methods

Fifty patients with histologically confirmed squamous cell carcinoma of the head and neck, treated with conformal RT, and who underwent pre- and post-treatment thyroid function tests were included in the study. All patients were euthyroid before treatment. The thyroid gland was contoured retrospectively in the approved RT plan. The volume of the contoured thyroid gland and the thyroid dosimetric parameters (Dmean, Dmax, Dmin, and V30-V60) were recorded. Pre- and post-treatment thyroid function test results were compared to look for the incidence of hypothyroidism, and dosimetric data were compared to establish a relation with the incidence of hypothyroidism.

Results

The median age was 53 years. Most patients had oral cavity primaries (60%), stage III/IVA disease (86%), and received definitive (64%) or adjuvant (36%) RT. Two-thirds underwent concurrent chemotherapy. After a median follow-up of four months, 24% developed hypothyroidism, with a median onset of 16 weeks post-treatment. Pharyngeal primary and concurrent chemotherapy were significant risk factors. Dosimetric analysis revealed Dmean > 57 Gy, V55 > 80%, and V60 > 37% as predictors of hypothyroidism.

Conclusion

Pharyngeal cancers, concurrent chemoradiotherapy, and higher thyroid doses significantly increase the risk of hypothyroidism. Optimizing RT planning is essential to minimize thyroid toxicity, particularly in high-risk patients.

## Introduction

Head and neck squamous cell carcinoma (HNSCC) encompasses a variety of malignancies, including cancers of the lip, oral cavity, nasopharynx, oropharynx, hypopharynx, larynx, nose, and paranasal sinuses. These cancers are notably prevalent in India, where they represent approximately 17.13% of all cancers, with a higher incidence in males. Globally, head and neck cancers (HNC) are the seventh most common cancers, accounting for 4.6% of cases [1].

For early-stage HNSCC, treatment typically involves single-modality therapies, such as surgery or radiation [2]. However, for more advanced diseases, concurrent chemoradiotherapy (CCRT) is often preferred due to its improved treatment outcomes [3]. A major challenge in treating advanced HNSCC is the risk of radiation-induced thyroid dysfunction, as the thyroid is often included in the radiation field [4]. Radiation to the neck region can lead to hypothyroidism, either subclinical (elevated thyroid-stimulating hormone (TSH) with normal free thyroxine (fT4)) or overt (high TSH, low fT4, and symptoms such as fatigue, cold intolerance, and weight gain) [5]. Though radiation-induced clinically overt hypothyroidism occurs within five years and manifests as late toxicities, subclinical hypothyroidism can occur as early as zero to three months [6].

Studies show that almost 50% of HNC patients who receive radiation to the neck develop some form of hypothyroidism. The mechanisms behind this include radiation-induced vessel damage, autoimmune reactions, and capsule fibrosis. Factors influencing the development of hypothyroidism include female sex, younger age, smaller thyroid volume, the addition of surgery, and the combination of chemotherapy with radiation [7,8]. Additionally, the irradiated volume of the thyroid gland, the extent of surgery, radiation dose, and duration of follow-up are important predictors for the development of hypothyroidism [9]. Modern radiotherapy (RT) techniques such as intensity-modulated radiation therapy (IMRT) and three-dimensional conformal radiation therapy (3DCRT) aim to minimize damage to healthy tissues, but even these methods do not completely eliminate the risk of thyroid complications [10].

Although no universal dose constraints are established for thyroid protection, several studies suggest that the volume of the thyroid receiving more than 30 Gy (V30), 45 Gy (V45), or 50 Gy (V50) is a significant predictor of hypothyroidism [4,11-15]. Moreover, the biological effect of concurrent chemotherapy, which sensitizes tissues to radiation, can further escalate the radiation dose to the thyroid, potentially exacerbating thyroid dysfunction [16,17].

The purpose of this study was to evaluate the incidence of biochemical hypothyroidism in HNC patients who received radiation therapy to the neck region and to identify a dose-volume histogram parameter that can independently predict post-radiation thyroid dysfunction at a regional cancer center in India.

## Materials and methods

Patients

Patients with histopathologically proven HNC who received RT to the head and neck region using a conformal technique between November 2022 and November 2023 were selected for the study and included for analysis according to the inclusion and exclusion criteria. Newly diagnosed, non-metastatic, previously untreated patients aged between 20 and 70 years with an Eastern Cooperative Oncology Group (ECOG) performance status (PS) of 0-2 and thyroid function test reports available at pre-treatment and during post-treatment follow-ups were included in the study. Patients with previously diagnosed thyroid disorders, cancer involving the thyroid gland, or a history of surgery involving complete or partial removal of the thyroid gland for the current disease or other comorbidities were excluded. A total of 50 patients were analyzed.

Treatment

All patients received treatment according to the standard institutional protocol. Eligible patients underwent RT to the face and neck region, delivered using the volumetric modulated arc therapy (VMAT) technique and image guidance on a linear accelerator (Elekta Synergy, Stockholm, Sweden) owned by the institute. Computed tomography (CT) simulation was performed for every case, and the resulting image data were transferred to the Monaco Treatment Planning System (TPS) for contour delineation of various target volumes. Standard contouring guidelines were followed in all cases for the delineation of primary and nodal targets, as well as organs at risk (OARs).

High-risk planning target volume (PTV) was prescribed either 66 Gy in 30 fractions or 69.96 Gy in 33 fractions (2.2 Gy or 2.12 Gy per fraction). The intermediate-risk PTV was prescribed a dose of either 60 Gy in 30 fractions or 59.4 Gy in 33 fractions (2 Gy or 1.8 Gy per fraction), and the low-risk PTV was prescribed a dose of either 54 Gy in 30 fractions or 54.12 Gy in 33 fractions (1.8 Gy or 1.64 Gy per fraction). Dose constraints for OARs were prescribed considering the dose per fraction based on the linear-quadratic model and calculated biologically effective dose (BED). 

Patients eligible for CCRT with normal auditory and renal function received weekly chemotherapy with cisplatin (40 mg/m²) for five to seven cycles. For patients with moderate-to-severe bilateral sensorineural hearing loss, carboplatin (AUC 2) was administered via intravenous infusion as an alternative to cisplatin.

Different parameters of the thyroid gland noted

The thyroid gland, located in the anteroinferior portion of the larynx, was contoured in all patients using appropriate imaging settings (window: 190-200 and level: 85-90) in the simulation CT retrospectively, and the volume of the contoured thyroid gland was recorded.

In the approved plan (which was delivered to the patients), the following dosimetric parameters were recorded: mean thyroid dose (Dmean), maximum and minimum thyroid doses (Dmax & Dmin), and percentage of thyroid gland volume receiving more than 30 Gy, 40 Gy, 45 Gy, 50 Gy, 55 Gy, and 60 Gy (V30, V40, V45, V50, and V60). These dosimetric data were analyzed.

Evaluation of thyroid function

As per standard institutional protocol, all patients were monitored weekly for any radiation- or chemotherapy-related toxicities and managed accordingly during the period of RT. The first follow-up visit occurred four weeks after treatment completion, followed by subsequent visits as per standard follow-up protocols. All eligible patients had thyroid profile reports available in each follow-up. Pre- and post-treatment TSH, T3, and T4 data were noted and evaluated to check for hypothyroidism, which was defined as a TSH value exceeding 5 μIU/mL. All thyroid function tests were conducted at the same laboratory, which is owned by the treating institute.

**Figure 1:**
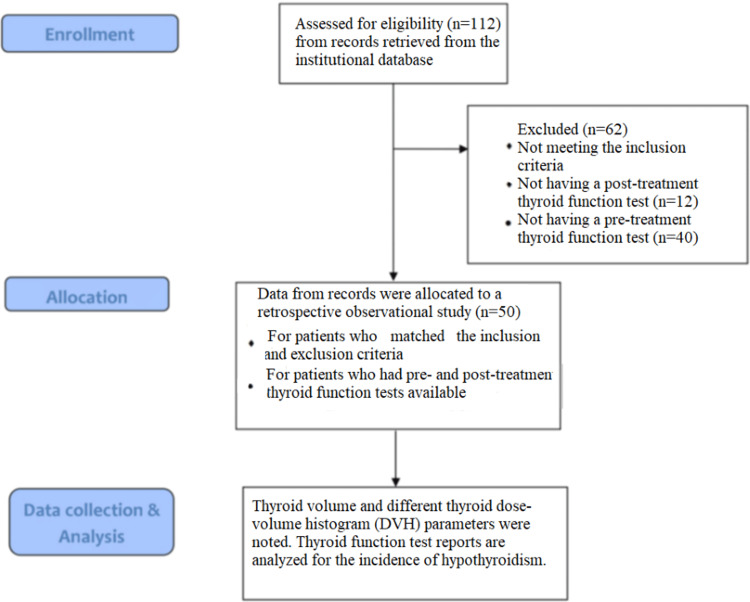
Flowchart depicting the methodology

Figure 1 depicts the flowchart of the methodology for the study.

Statistical analysis

Data were analyzed using Statistical Product and Service Solutions (SPSS, version 23.0; IBM SPSS Statistics for Windows, Armonk, NY). The analysis evaluated various independent variables, such as age, sex, disease stage, tumor site, treatment type, radiation dose, chemotherapy status, thyroid volume, and dose-volume histogram (DVH) parameters, with the outcome being post-RT thyroid function status (euthyroid or hypothyroid). Descriptive statistics were used to summarize continuous variables as means with standard deviations and categorical variables as counts and percentages.

To compare nominal variables between euthyroid and hypothyroid groups, chi-square (χ²) or Fisher’s exact tests were used. For continuous data, differences between means were assessed using Student’s t-test (for equal variances) or Welch’s t-test (for unequal variances), with Levene’s test checking for variance equality. Some continuous variables were dichotomized using the median as a cutoff and analyzed with the chi-square test. A p-value of <0.05 was considered statistically significant.

## Results

Patient characteristics

This study involved 50 patients, of which 74% were male (37 patients) and 26% were female (13 patients), showing a significantly higher proportion of male patients. The male-to-female ratio was 2.8:1. The patients' ages ranged from 32 to 70 years, with a median age of 53 years. The study included 16% of patients under 40 years, with the remaining participants fairly distributed across age groups: 41-50 years (28%), 51-60 years (26%), and 61-70 years (30%). The majority of patients had oral cavity cancers (60%), while nasopharyngeal and oropharyngeal cancer patients made up 16% and 14%, respectively. Laryngeal and hypopharyngeal carcinoma patients were fewer (three and two patients, respectively). Most patients presented with stage III (40%) and stage IVA (46%) disease.

Regarding treatment, 58% of patients received definitive CCRT, 28% received adjuvant RT, 8% received adjuvant CCRT, and 6% received definitive RT. Most patients (56%) were prescribed a total dose of 66 Gy for high-risk PTV. Eighteen patients (36%) received postoperative treatment, with 14 patients receiving RT at 60 Gy, and four receiving CCRT at 66 Gy. Only eight patients with nasopharyngeal carcinoma received 70 Gy. A total of 66% of patients received concurrent chemotherapy, significantly more than those who did not (Table 1).

**Table 1:** Patient characteristics

Patient characteristics		Value (out of 50)	Percentage
Age Group			
	30-40 years	8	16
	41-50 years	14	28
	51-60 years	13	26
	61-70 years	15	30
Gender			
	Male	37	74
	Female	13	26
Site			
	Oral cavity	30	60
	Oropharynx	7	14
	Nasopharynx	8	16
	Hypopharynx	2	4
	Larynx	3	6
Stage			
	I	1	2
	II	6	12
	III	20	40
	IVA	23	46
Treatment Received			
	Definitive CCRT	29	58
	Adjuvant CCRT	4	8
	Adjuvant RT	14	28
	Definitive RT	3	6
Concurrent Chemotherapy			
	Yes	33	66
	No	17	34

Thyroid dosimetry

In terms of dosimetric analysis, the maximum, mean and median thyroid doses (Dmax, Dmean, Dmin, respectively) were 63.07 Gy, 50.81 Gy, and 31.94 Gy, with corresponding standard deviations. The percentage of thyroid volume receiving at least 30 Gy and 40 Gy was 100%, with lesser percentages for higher doses (97.01% for V45, 93.35% for V50, 78.02% for V55, and 36.45% for V60) (Table 2).

**Table 2:** Dosimetric characteristics

Dosimetric characteristics of the thyroid	Mean ± SD	Median
Dmean (Gy)	50.81±14.15	56.75
Dmax (Gy)	63.07±10	65.06
Dmin (Gy)	31.94±19.29	38.5
V30 (%)	84.44±24.44	100
V40 (%)	81.95±26.60	100
V45 (%)	80.42±27.22	97.01
V50 (%)	77.75±27.54	93.35
V55 (%)	64.51±33.95	78.02
V60 (%)	36.73±32.83	36.45

Incidence of hypothyroidism and its relation with patient demographics and treatment characteristics

**Figure 2:**
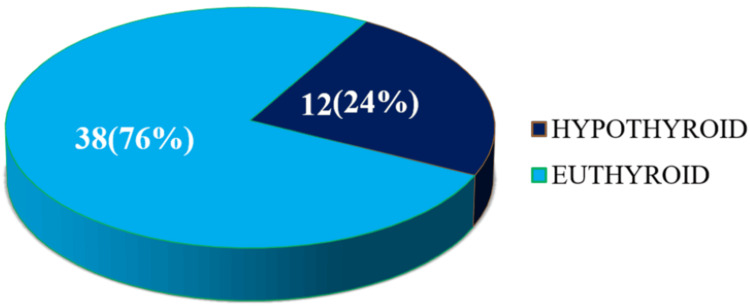
Incidence of hypothyroidism after median follow-up of four months

After a median follow-up of four months, 12 patients (24%) developed hypothyroidism, with a median onset of four months (range: 4-28 weeks) (Figure 2). Only one patient had clinical hypothyroidism, while the rest were subclinical.

The incidence of hypothyroidism was higher in patients aged 41-50 years (50%), compared to those in the 30-40 years group (8.3%). Gender did not significantly affect the development of hypothyroidism (p=0.48). Hypothyroidism was more common in pharyngeal cancers compared to oral cavity cancers (p=0.003), with 91.7% of hypothyroid patients having received definitive CCRT. However, this difference was not statistically significant (p=0.073). The study also found a higher incidence of hypothyroidism in patients receiving higher radiation doses. Hypothyroidism occurred in 7.1% of patients who received 60 Gy, 28.6% of those who received 66 Gy, and 37.5% of those who received 70 Gy, but these differences were not statistically significant (p=0.211). Patients who received concurrent chemotherapy had a significantly higher rate of hypothyroidism (33.3% vs. 5.9%, p=0.039) (Table 3).

**Table 3:** Relation of hypothyroidism with different demographic parameters

Patient characteristics		Euthyroid (out of 50)	Hypothyroid (out of 50)	P-value
Age Group				
	30-40 years	7	1	
	41-50 years	8	6	0.3
	51-60 years	10	3	
	61-70 years	13	2	
Gender				
	Male	27	10	0.42
	Female	11	2	
Site				
	Oral cavity	27	3	
	Oropharynx	4	3	0.003
	Nasopharynx	4	4	
	Hypopharynx	0	2	
	Larynx	3	0	
Stage				
	I	1	0	
	II	6	0	0.373
	III	13	7	
	IVA	18	5	
Treatment Received				
	Definitive CCRT	18	11	
	Adjuvant CCRT	4	0	0.073
	Adjuvant RT	13	1	
	Definitive RT	3	0	
Concurrent Chemotherapy				
	Yes	22	11	0.039
	No	16	01	
Total Dose Received				
	70 Gy	5	3	
	66 Gy	20	8	0.211
	60 Gy	13	1	

Thyroid dosimetry and incidence of hypothyroidism

Regarding radiation doses, those who developed hypothyroidism received higher mean doses of Dmean, Dmax, and Dmin compared to those who remained euthyroid, with only the difference in Dmean being statistically significant (p=0.016). Similarly, the patients who developed hypothyroidism had significantly higher volumetric dose distributions, particularly for V30, V40, V45, and V50 (p<0.05) (Table 4).​​​​

**Table 4:** Relation of hypothyroidism with different dosimetric parameters V30, V40, V45, V50=Dose-volume parameters

Dosimetric parameters		Euthyroid (Mean±SD)	Hypothyroid (Mean±SD)	P-value
Dmean		48.82±15.22	57.11±7.51	0.016
Dmax		62.39±11.07	65.21±5.15	0.4
Dmin		29.30±19.80	40.31±15.43	0.57
V30 (%)		81.16±26.47	94.83±12.23	0.018
V40 (%)		78.38±28.47	93.26±15.52	0.027
V45 (%)		76.68±29.03	92.25±16.25	0.025
V50 (%)		74.24±29.51	88.89±16.51	0.037

**Figure 3:**
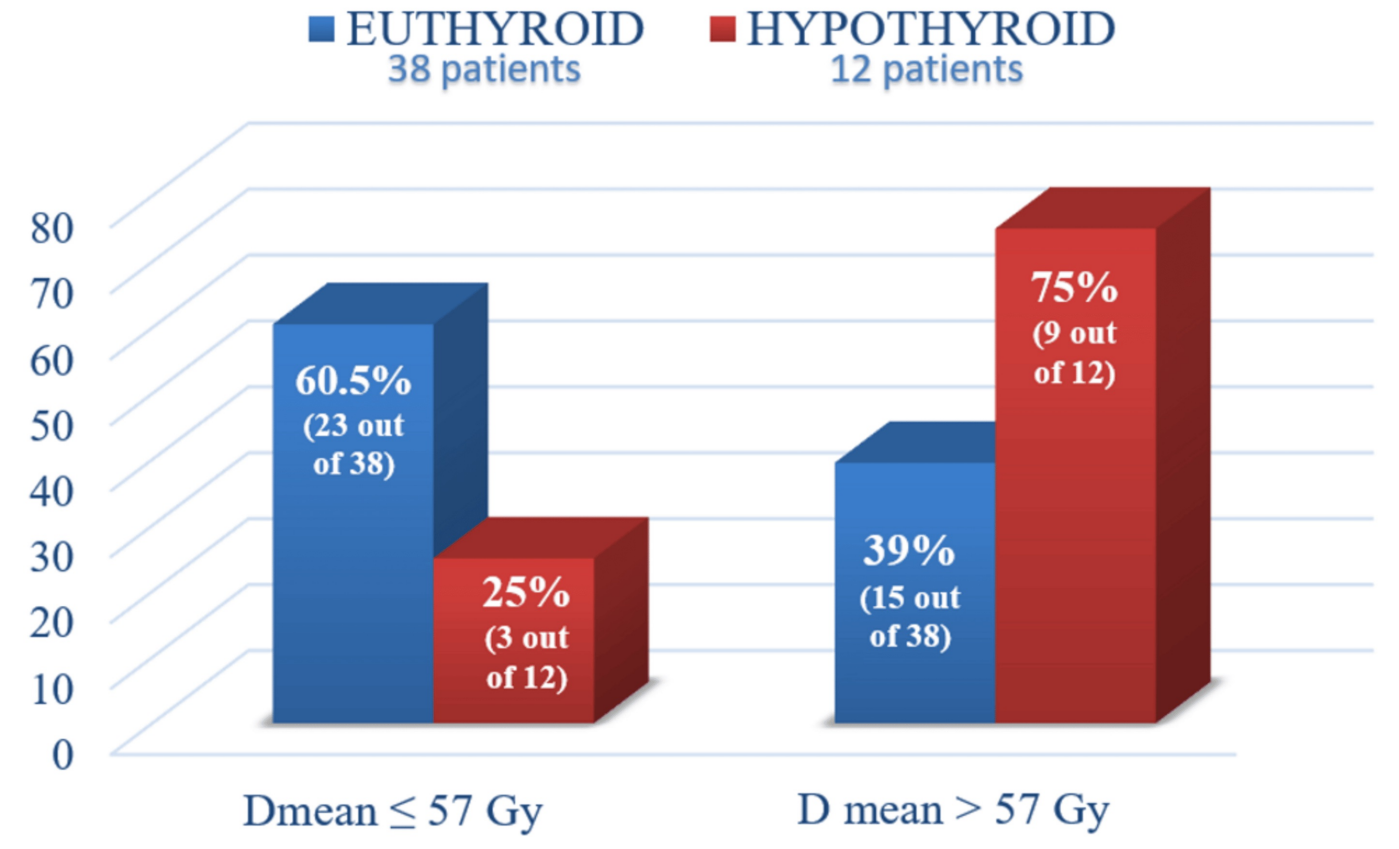
Bar Diagram showing incidence of hypothyroidism according to Dmean value (p=0.032)

The study also revealed that a Dmean > 57 Gy significantly increased the risk of hypothyroidism (p=0.032) (Figure 3).

**Figure 4:**
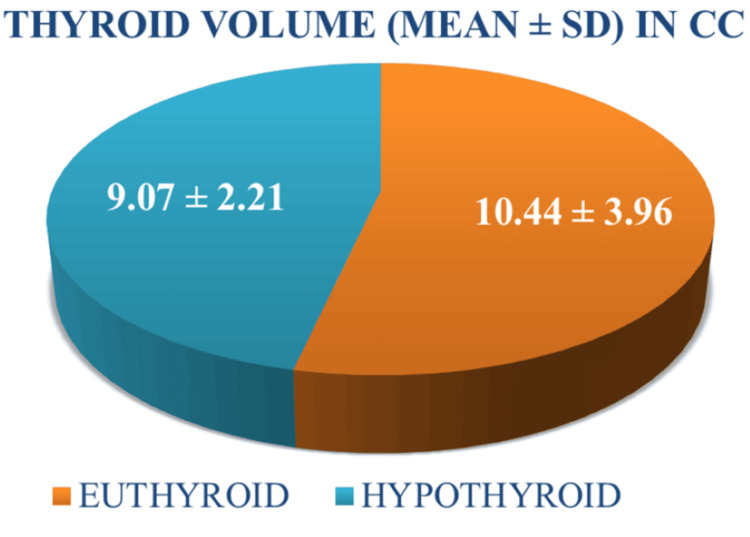
Relation of thyroid volume with hypothyroidism incidence (p=0.262) CC=Cell carcinoma

Thyroid gland volume did not show a significant difference between the groups (p=0.262) (Figure 4).

Among the patients who remained euthyroid, 57.9 % had V55 of thyroid gland ≤ 80 %. On the contrary, 75% of the patients who developed hypothyroidism had thyroid V55 > 80% in this study. This difference was statistically significant (p=0.047). Similar hypothyroidism incidence was found at a cutoff of 37% for V60 (p=0.032).

## Discussion

This study evaluated 50 patients with HNSCC treated between November 2022 and November 2023 using the VMAT technique. Patients included in the study had both pre-treatment and post-treatment thyroid profile reports available. The ages ranged from 32 to 70 years, with a mean age of 52.44 years (standard deviation=11.21). The majority of patients (74%) were male, consistent with previous findings that HNSCC is more common in males in India [1,18]. Age distribution was as follows: 16% under 40 years, 28% between 41 and 50 years, 26% between 51 and 60 years, and 30% between 61 and 70 years. These demographics align with observations by Sachdev et al. and Diaz et al. [14,19].

Most patients (60%) had oral cavity cancers, while nasopharyngeal (16%) and oropharyngeal (14%) cancers were less frequent. A small percentage of patients had laryngeal (6%) or hypopharyngeal (4%) cancers, consistent with studies showing oral cavity cancers as the most prevalent HNC in India [20]. Late-stage disease was predominant, with 40% of patients presenting with stage III and 46% with stage IVA, aligning with studies by Alterio et al. and Diaz et al. [7,19]. Definitive chemoradiotherapy (58%) was the primary treatment modality, with 28% receiving adjuvant RT and 6% receiving definitive RT alone. Concurrent chemotherapy, predominantly with cisplatin or carboplatin, was administered to 66% of patients, consistent with treatment protocols described by Kim et al. and Lo Galbo et al. [4,21].

The primary focus was on hypothyroidism incidence after treatment with radiation. During a median follow-up of four months (range: 10-28 weeks), 24% of patients developed hypothyroidism, with a median onset of four months. Of these, 2% had clinical hypothyroidism, while the remainder had subclinical hypothyroidism, a rate consistent with prior studies reporting hypothyroidism rates of 23%-42% following RT [21-23].

Several factors were analyzed for their association with hypothyroidism, including age, gender, primary tumor site, stage of disease, treatment type, radiation dose, and chemotherapy. The study found no significant correlation between age and the development of hypothyroidism, consistent with the findings of Kim et al. and Fujiwara et al. [4,24]. Similarly, gender did not significantly affect the incidence of hypothyroidism, despite a higher proportion of male patients. The low proportion of female patients in the study may have limited the ability to detect gender-related differences.

A significant difference in hypothyroidism incidence was observed based on tumor location. Hypothyroidism was more common in patients with pharyngeal cancers (nasopharyngeal, oropharyngeal, and hypopharyngeal) compared to oral cavity cancers. Specifically, 50% of nasopharyngeal cancer patients and 42.9% of oropharyngeal cancer patients developed hypothyroidism, whereas 90% of oral cavity cancer patients and all hypopharyngeal cancer patients remained euthyroid. This finding aligns with previous studies by Zhou et al., Huang et al., and Kamal et al. [15,25,26].

The cancer stage did not significantly correlate with hypothyroidism development. All stage I and II patients remained euthyroid, 58.3% of hypothyroid patients were stage III, and 41.7% were stage IV, contrasting findings by Kamal et al. [26] that advanced stages may predict hypothyroidism risk. However, CCRT significantly increased hypothyroidism risk (33.3% vs. 5.9%, p=0.039), supported by studies indicating chemotherapy-enhanced radiosensitivity as a contributing factor [16,27].

Although higher radiation doses were associated with increased hypothyroidism incidence, this difference was not statistically significant. Similarly, previous studies by Koc et al. and Tell et al. [6,8] found no strong correlation between radiation dose and hypothyroidism. In contrast, some studies, such as those by Lo Galbo et al. and Mercado et al. [21,28], suggest that higher doses, particularly those exceeding 65 Gy, increase the risk. Different indications for neck irradiation (whether due to node positivity, extranodal extension, or elective treatment) result in variations in the dose to nodal regions and, consequently, to the thyroid gland, which has not been evaluated as a cofactor for the incidence of hypothyroidism in this study. This can explain why the total prescribed dose had a non-significant effect on the incidence of hypothyroidism. Dosimetric analysis showed that the mean dose to the thyroid (Dmean) was a significant predictor of hypothyroidism (p=0.016). Patients receiving Dmean > 57 Gy had a significantly higher hypothyroidism incidence (37.5% vs. 11.5%, p=0.032), consistent with findings from Kim et al., Chyan et al., and Boomsma et al. [4,13,29]. Dose-volume parameters, particularly V55 and V60, were also significant predictors, with hypothyroidism being more common in patients with V55 > 80% (p=0.047) and V60 > 37% (p=0.032), aligning with studies by Akgun et al. and Lee et al. [12,30].

Limitations

This study had certain limitations. First, the shorter follow-up duration resulted in a low incidence of clinical hypothyroidism. Since thyroid dysfunction is a late side effect of radiation, a longer follow-up period would likely have provided more comprehensive documentation of its overall incidence. Second, no dose constraints were applied to the thyroid gland during treatment planning, leading to higher radiation doses to the thyroid. Third, different indications for neck irradiation (whether due to node positivity, extranodal extension, or elective treatment) result in variations in the dose to nodal regions and, consequently, to the thyroid gland, which has not been evaluated as a cofactor for incidence of hypothyroidism in this study. This may have influenced the correlation between radiation-induced hypothyroidism and various thyroid dosimetric parameters, potentially differing from findings reported in published studies.

## Conclusions

This study underscores the intricate relationship between radiation dosimetry and the development of hypothyroidism in patients undergoing RT for HNC. The findings indicate that higher radiation doses to the thyroid gland and the use of concurrent chemotherapy significantly increase the risk of hypothyroidism. These results highlight the need for meticulous dosimetric planning and long-term monitoring of thyroid function, particularly in patients receiving high radiation doses or concurrent chemotherapy.

Since hypothyroidism can manifest as both an early and late sequela of treatment, thyroid function monitoring should begin immediately after the completion of RT in all HNC patients. The thyroid gland should be contoured during RT planning and ideally treated as an OAR. Although a universally accepted consensus on dose constraints for the thyroid gland is lacking, efforts should be made to minimize thyroid doses where feasible. Specifically, maintaining a thyroid Dmean ≤ 57 Gy, thyroid V55 ≤ 80%, and thyroid V60 ≤ 37%, without compromising the target volume dose, could help reduce the incidence of hypothyroidism. Further studies with larger cohorts and extended follow-up durations are necessary to validate the results and dose constraints identified in this study.
